# Polysaccharide of *Atractylodes macrocephala* Koidz (PAMK) Relieves Immunosuppression in Cyclophosphamide-Treated Geese by Maintaining a Humoral and Cellular Immune Balance

**DOI:** 10.3390/molecules23040932

**Published:** 2018-04-17

**Authors:** Wanyan Li, Sixuan Guo, Danning Xu, Bingxin Li, Nan Cao, Yunbo Tian, Qingyan Jiang

**Affiliations:** 1 Guangdong Provincial Key Laboratory of Animal Nutrition Control, College of Animal Sciences, South China Agricultural University, Guangzhou 510642, China; lwanyan88@126.com; 2 Guangdong Province Key Laboratory of Waterfowl Healthy Breeding, Guangzhou 510225, China; g765380373@126.com (S.G.); xdanning212@126.com (D.X.); libingxin212@126.com (B.L.); caonan870405@126.com (N.C.); 3 College of Animal Science & Technology, Zhongkai University of Agriculture and Engineering, Guangzhou 510225, China

**Keywords:** polysaccharide of *Atractylodes macrocephala* Koidz, cyclophosphamide, spleen, cellular immunity, humoral immunity, principal component analysis

## Abstract

Polysaccharide of *Atractylodes macrocephala* Koidz (PAMK) has been well recognized as an immune enhancer that can promote lymphocyte proliferation and activate immune cells. The purpose of this study was to evaluate the effects of PAMK on humoral and cellular immune functions in immunosuppressed geese. Geese of the Control group were provided with normal feed, the PAMK group was provided with 400 mg·(kg body weight)^−1^ PAMK, the cyclophosphamide (CTX) group was injected with 40 mg·(kg body weight)^−1^ cyclophosphamide, while the CTX+PAMK group received the combination of PAMK and CTX. Spleen development and percentages of leukocytes in peripheral blood were examined. Principal component analysis was conducted to analyze correlations among humoral and cellular immune indicators. The results showed that PAMK alleviated the damage to the spleen, the decrease in T- and B-cell proliferation, the imbalance of leukocytes, and the disturbances of humoral and cellular immunity caused by CTX. Principal component analysis revealed that the relevance of humoral-immunity-related indicators was greater, and the CTX+PAMK group manifested the largest difference from the CTX group but was close to the Control group. In conclusion, PAMK alleviates the immunosuppression caused by CTX in geese, and the protective effect on humoral immunity is more obvious and stable.

## 1. Introduction

Polysaccharide of *Atractylodes macrocephala* Koidz (PAMK), the main active ingredient of *Atractylodes macrocephala*, has been confirmed to regulate immune function by antioxidant and antistress effects and to enhance an immune response [1,2]. In cellular immunity, PAMK mainly promotes T-lymphocyte proliferation and cytokine secretion. It has been reported that PAMK significantly enhances peripheral-blood T-lymphocyte proliferation (in humans, mice, and chickens) and spleen T-lymphocyte proliferation (in mice and chickens), induces T lymphocytes to enter S and G2/M phases, and effectively elevates the percentages of CD4+ and CD8+ T cells [1,3,4]. Besides, PAMK significantly increases the serum levels of IFN-γ, TNF-α, and IL-6 in mice and the serum levels of IL-2, IL-6, IL-4, TNF-α, and IFN-γ in chickens [3,4]. However, Xu [2,5] has reported that PAMK decreases the serum levels of IL-2 and IFN-γ increased by heat stress and significantly increases the protein levels of IFN-γ decreased by heat stress in chickens. Therefore, the effects of PAMK on cytokines are complex and have not yet been clearly reported. Research into the effects of PAMK on humoral immunity is scarce. It has been reported only that feeding with PAMK significantly increases the total IgG level in the serum of hen egg-white lysozyme (HEL)-immunized mice and induces an antigen-specific humoral immune response [3]. Besides, PAMK and selenylated PAMK can improve the antibody titers of chickens inoculated with the Newcastle disease (ND) vaccine [4].

When it comes to other polysaccharides from traditional Chinese medicine, such as *Astragalus polysaccharides*, *Schisandra chinensis* (Turcz.), and curcuminoids, they have been proved to modulate immunity through the TLR4 signaling pathway and eventually to participate in the regulation of nuclear factor kappa B (NF-κB) activation [6,7,8,9,10]. However, reports about the mechanism of PAMK-mediated regulation of immune function are few. It has been demonstrated that PAMK modulates macrophage activities by inducing inhibitor kappa B (IκB) degradation and activation of NF-κB by nuclear translocation of p65 [11]. Besides, atractylenolide I (AO-I), another major bioactive component from *Atractylodes macrocephala*, has been shown to protect mice from acute lung injury induced by lipopolysaccharide (LPS) via inhibition of Toll-like receptor 4 (TLR4) expression and NF-κB activation [7].

Reports about the immunomodulatory effects of PAMK mainly deal with mice and chickens. There is no report on the effects of PAMK on immunosuppression in geese. To further elucidate the influence of PAMK on immune function, we focused on the effect of PAMK on humoral and cellular immunity of geese immunosuppressed by cyclophosphamide (CTX). Principal component analysis was performed to compare the effects of PAMK on humoral and cellular immunity.

## 2. Results

### 2.1. PAMK Alleviated CTX-Induced Spleen Damage

**Figure 1:**
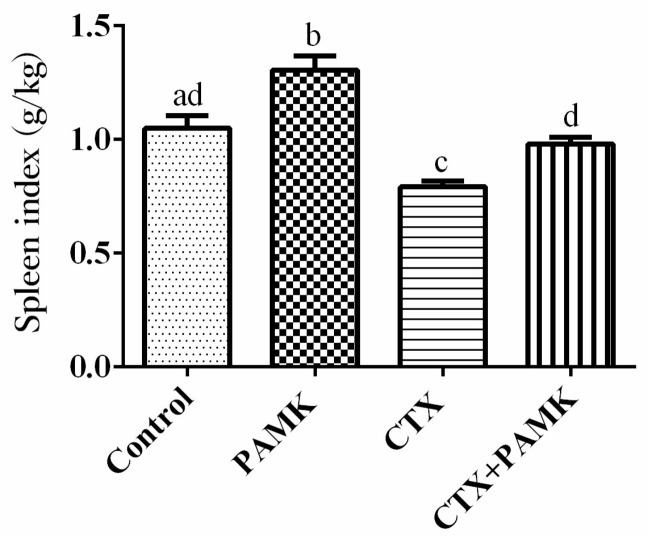
Effects of polysaccharide of *Atractylodes macrocephala* Koidz (PAMK) on the spleen index decreased by cyclophosphamide (CTX). The data are expressed as the mean ± SD, *n* = 20. Means with different letters represent statistically significant differences (*p* < 0.05); the bars with the same letter are not significantly different (*p* > 0.05).

Results on the spleen index showed that CTX had a significant inhibitory effect on development of the spleen (*p <* 0.05), indicating that the immunosuppressive model had been successfully created (Figure 1). However, PAMK significantly increased the spleen index of normal (*p* < 0.05) and CTX-treated (*p* < 0.05) geese. The spleen index in the CTX+PAMK group was significantly higher than that of the CTX group but did not reach the level of the Control group.

**Figure 2:**
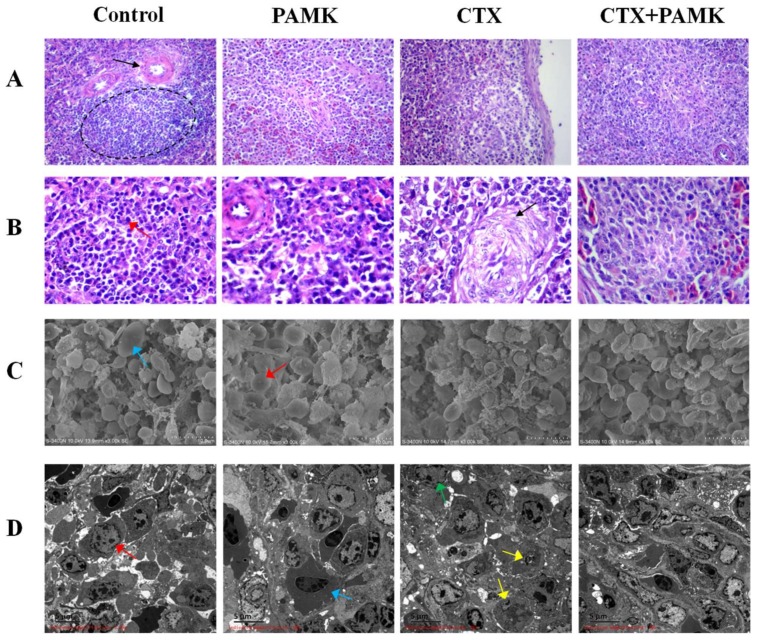
Effects of PAMK on histology and ultramicroscopic morphology of the spleen of geese treated with CTX. (**A**) Hematoxylin and eosin (HE) staining of the spleen (400×); (**B**) HE staining of the spleen (1000×); (**C**) Scanning electron microscopy (SEM; 3000×) of the spleen. (**D**) Transmission electron microscopy (TEM; 3000×) of the spleen. Circles indicate the periarterial lymphatic sheath (PLS), the black arrow points to the artery, the red arrow indicates a lymphocyte, the yellow arrow points to an apoptotic lymphocyte, the green arrow reveals in a lymphocyte that chromatin is concentrated on the nuclear membrane, and the blue arrow indicates an erythrocyte.

Hematoxylin and eosin (HE) staining revealed that the demarcation between the red pulp and white pulp of the goose spleen was not clear (Figure 2A). The Control group had a normal periarterial lymphatic sheath (PLS) and normal cell morphology. As compared with the Control group, the PLS in the PAMK group was smaller, and the white pulp area was larger, but there was no difference in cell morphology. Cells in the CTX group were arranged loosely, there was a large number of vacuoles, the structure of PLS was incomplete, and the number of lymphocytes significantly decreased. However, as compared with the CTX group, cells in the CTX+PAMK group were arranged densely, and the PLS was normal and showed no difference from the PAMK group. A high-magnification microscope was used to further examine the structure of the spleen (Figure 2B). Cells in the Control group were arranged regularly, and lymphocyte morphology was normal. Lymphocytes in the PAMK group were normal, the structure of arterioles was normal, and the morphology was the same as that in the Control group. Cells in the CTX group were arranged irregularly, lymphocyte differentiation was weak, nuclear and chromatin were loose, and there were more vacuolized cells. Endothelial cells and smooth muscle fibers of arteries manifested transparent degeneration. However, compared with the CTX group, the CTX+PAMK group had a greater number of regular lymphocytes.

In the scanning electron microscopy (SEM) images of the spleen (Figure 2C), the Control group had normal structure, normal cell morphology, no erythrocyte shrinkage, and lymphocytes were activated. The structure and cell morphology of the spleen in the PAMK group were similar to those in the Control group. The spleen of the CTX group had a loose structure, a small number of lymphocytes, and abnormal morphology; lymphocyte apoptosis was substantial, with blebbing of the cell surface; and erythrocytes underwent shrinkage. Morphology in the CTX+PAMK group was significantly better than that in the CTX group; only a small number of cells was in the early stages of apoptosis, and lymphocytes were in an inactive state.

Transmission electron microscopy (TEM) of the spleen indicated that lymphocytes in the Control group had normal nuclear mass and chromatin in a normal configuration (Figure 2D). The cell structure in the PAMK group was clear-cut, the distributions of chromatin and heterochromatin in the nucleus were uniform, and the structure of the cell membrane was clear-cut. The nuclei in the CTX group showed a tendency for shrinkage, and chromatin deficiency was obvious: heterochromatin decreased, and chromatin was concentrated on the nuclear membrane. It was found that there were greater numbers of apoptotic lymphocytes in the CTX group. Compared with the CTX group, the CTX+PAMK group manifested no other significant differences, but apoptotic lymphocytes significantly decreased in number.

### 2.2. PAMK Alleviated the CTX-Induced Leukocyte Ratio Imbalance

**Figure 3:**
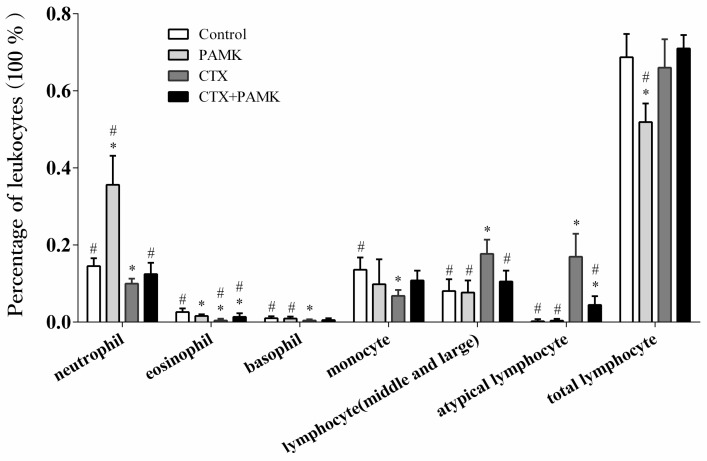
The effect of PAMK on the percentages of leukocytes in the peripheral blood of CTX-treated geese. Data are presented as the mean ± SD, *n* = 8. * *p* < 0.05, as compared with the Control group; # *p* < 0.05, as compared with the CTX group.

Analysis of the percentages of leukocytes (Figure 3) indicated that in the PAMK group, the percentage of neutrophils significantly increased (*p* < 0.05) and the percentages of eosinophils and lymphocytes significantly decreased (*p* < 0.05) as compared with the Control group, but PAMK had no significant effect on the percentages of other leukocytes (*p* > 0.05). Besides, the percentages of neutrophils, eosinophils, basophils, and monocytes in the CTX group were significantly lower than those in the Control group (*p* < 0.05); the percentages of atypical lymphocytes and lymphocytes (medium and large size) significantly increased (*p* < 0.05), while the percentage of lymphocytes showed no significant change (*p* > 0.05). Neutrophils, eosinophils, and monocytes perform phagocytosis of pathogens, all of them are important for a nonspecific immune response. Therefore, CTX may have an inhibitory effect on the nonspecific immune function of geese; thus, the immunosuppressive effect was obvious. However, the percentages of neutrophils and eosinophils significantly increased in the CTX+PAMK group as compared with the CTX group. Among leukocytes, the percentages of neutrophils, basophils, and monocytes showed no significant difference with the Control group (*p* > 0.05), while the percentage of eosinophils was still significantly lower (*p* < 0.05). In addition, the percentages of atypical lymphocytes and lymphocytes (medium and large size) in the CTX+PAMK group were significantly lower than those in the CTX group, but the percentage of atypical lymphocytes was still higher than that in the Control group. The proportion of each leukocyte type in the CTX+PAMK group was closer to that in the Control group. It can be speculated that PAMK can alleviate the CTX-induced leukocyte ratio imbalance.

### 2.3. PAMK Alleviated CTX-Induced Humoral-Immunity Dysfunction

**Figure 4:**
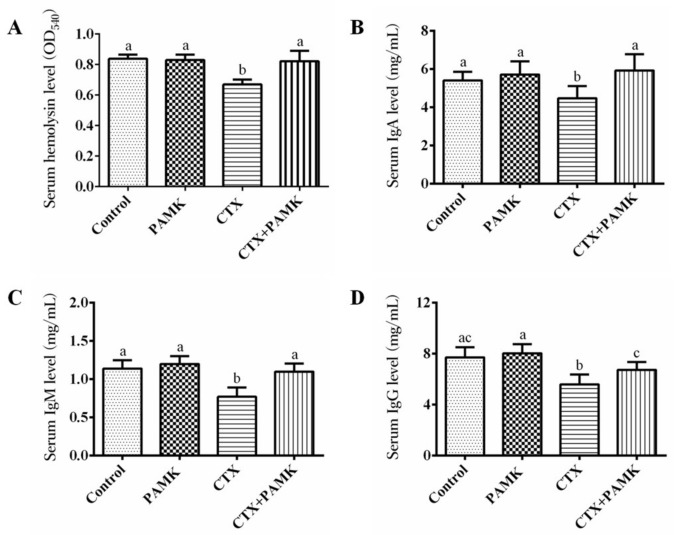
Effects of PAMK on hemolysin and immunoglobulin in the serum of geese treated with CTX. (**A**) Serum hemolysin levels (optical density at 540 nm; OD_540_). (**B**) Serum IgA levels. (**C**) Serum IgM levels. (**D**) Serum IgG levels. The data are expressed as the mean ± SD, *n* = 8. Means with different letters represent statistically significant differences (*p* < 0.05); the bars with the same letter are not significantly different (*p* > 0.05).

Separation of serum and analysis of hemolysin and immunoglobulin levels were performed to assess the ability of geese to produce antibodies (Figure 4). CTX simultaneously reduced the concentration of hemolysin (*p <* 0.05), IgA (*p <* 0.05), IgM (*p <* 0.05), and IgG (*p <* 0.05) in serum, further indicating that CTX can cause humoral-immunity dysfunction. Although PAMK did not significantly increase the levels of hemolysin, IgA, IgM, and IgG in the serum of normal geese (*p* > 0.05), it alleviated the inhibitory effect of CTX and significantly attenuated the decrease in levels of hemolysin (*p <* 0.05), IgA (*p <* 0.05), IgM (*p <* 0.05), and IgG (*p <* 0.05). The levels of hemolysin, IgA, IgM, and IgG in the serum of the CTX+PAMK group revealed no significant difference with the Control group (*p* > 0.05). These data suggested that PAMK had no significant effects on the humoral immunity of geese but significantly alleviated the humoral-immunity dysfunction induced by CTX.

**Figure 5:**
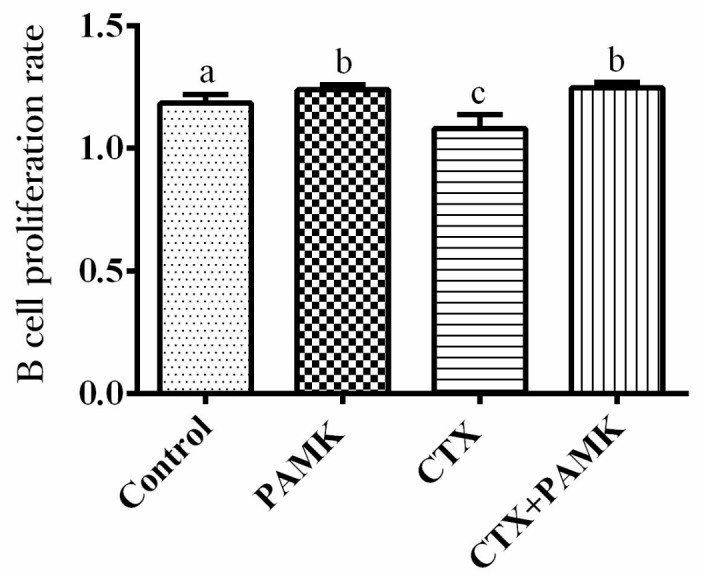
Effects of PAMK on proliferation of B cells from geese treated with CTX. The B-cell proliferation rate. The data are expressed as the mean ± SD, *n* = 8. Means with different letters represent statistically significant differences (*p <* 0.05); the bars with the same letter are not significantly different (*p* > 0.05).

Spleen lymphocytes of each group were isolated and treated with LPS to activate B-cell proliferation; we measured the proliferation rate to evaluate the functional potential of B cells (Figure 5). The results suggested that the in vitro proliferation rate of B cells from geese treated with CTX significantly decreased (*p <* 0.05), but in the PAMK-treated group, it significantly increased (*p <* 0.05). In addition, the proliferation rate of B cells in the CTX+PAMK group was significantly higher than that in the CTX group (*p <* 0.05) and recovered to the same level as in the PAMK group, indicating that PAMK can attenuate the inhibitory effect of CTX.

### 2.4. PAMK Alleviates CTX-Induced Cellular-Immunity Dysfunction

**Figure 6:**
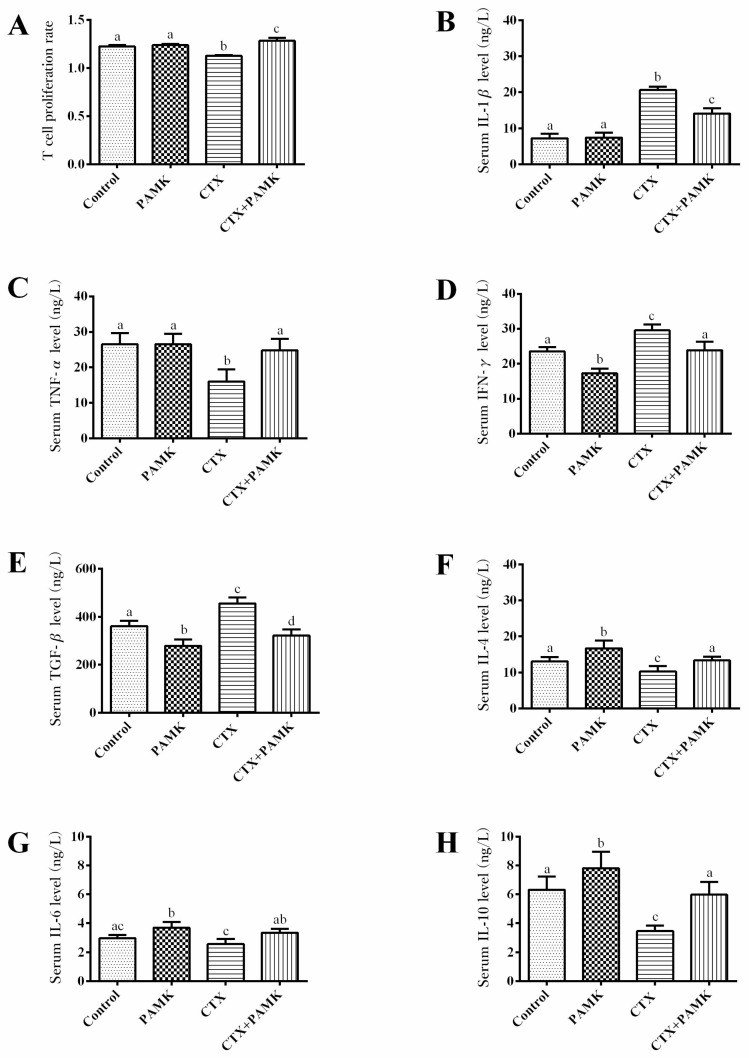
Effects of PAMK on the cellular immunity of geese treated with CTX. (**A**) T-cell proliferation rates; (**B**) Serum IL-1β levels; (**C**) Serum TNF-β levels; (**D**) Serum IFN-γ levels; (**E**) Serum TGF-β levels; (**F**) Serum IL-4 levels; (**G**) Serum IL-6 levels; (**H**) Serum IL-10 levels. The data are expressed as the mean ± SD, *n* = 8. Means with different letters represent statistically significant differences (*p <* 0.05); the bars with the same letter are not significantly different (*p* > 0.05).

Spleen lymphocytes of each group were isolated and treated with phytoagglutinin (PHA) to activate T-cell proliferation; we measured the proliferation rate to evaluate the functional potential of T cells. Consistent with the results on B cells, the in vitro proliferation rate of T cells from geese treated with CTX significantly decreased (*p <* 0.01) and was significantly lower than that in the CTX+PAMK group (*p <* 0.01; Figure 6A). In contrast to B cells, the proliferation rate of T cells in the PAMK group manifested no significant difference from the Control group (*p* > 0.05). This finding suggested that PAMK had no significant effect on normal T cells but attenuated the inhibitory effect of CTX and increased their proliferation to a level higher than that in the Control group.

Proinflammatory cytokines (IL-1β, TNF-α, and IFN-γ) and anti-inflammatory cytokines (TGF-β, IL-4, IL-6, and IL-10) in serum were quantified to study cellular immune function (Figure 6B–H). Treatment with PAMK significantly increased the levels of IL-4, IL-6, and IL-10 (*p <* 0.05), significantly decreased the level of IFN-γ and TGF-β (*p <* 0.05), but had no effects on the levels of IL-1β and TNF-α (*p* > 0.05). CTX had the opposite effects, i.e., significantly decreased the levels of TNF-α, IL-4, and IL-10 (*p <* 0.05); significantly increased the levels of IL-1β, IFN-γ, and TGF-β (*p <* 0.05); but had no effect on the concentration of IL-6 (*p* > 0.05). As compared to the CTX group, levels of TNF-α, IL-4, IL-6, and IL-10 significantly increased (*p <* 0.05), but levels of IL-1β, IFN-γ, and TGF-β significantly decreased (*p <* 0.05) in the CTX+PAMK group. It was demonstrated that the serum levels of cytokines in the CTX+PAMK group were closer to those in the Control group, suggesting that CTX has a pro-inflammatory effect, but PAMK has an anti-inflammatory effect and can even alleviate the inflammation induced by CTX.

**Figure 7:**
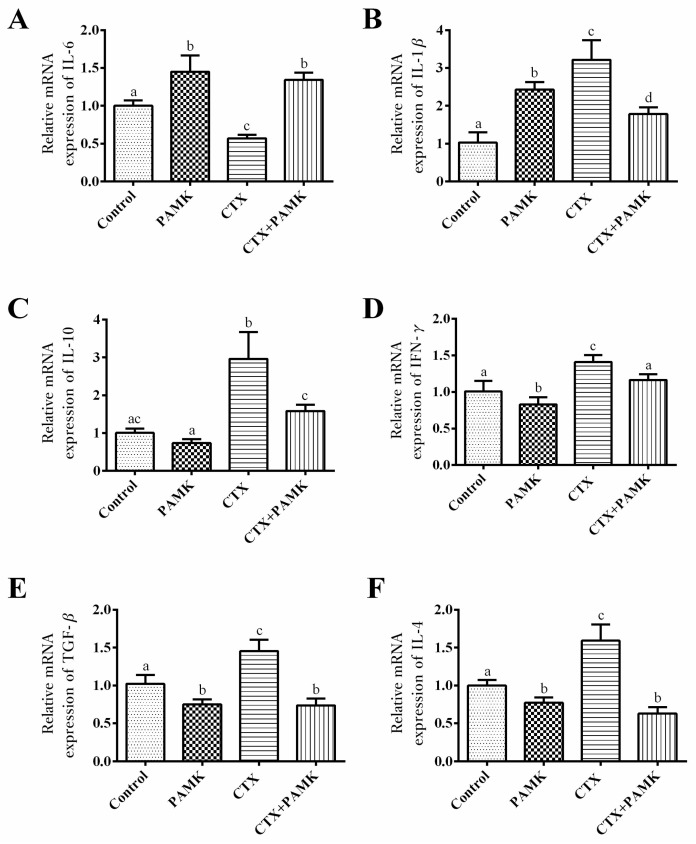
Effects of PAMK on the cellular immunity of geese treated with CTX. (**A**) Relative mRNA expression of IL-6; (**B**) Relative mRNA expression of IL-1β; (**C**) Relative mRNA expression of IL-10; (**D**) Relative mRNA expression of IFN-γ; (**E**) Relative mRNA expression of TGF-β; (**F**) Relative mRNA expression of IL-4. The data are expressed as the mean ± SD, *n* = 8. Means with different letters represent statistically significant differences (*p <* 0.05); the bars with the same letter are not significantly different (*p* > 0.05).

Total RNA was extracted from a spleen to measure the transcription levels of cytokines in the spleen (Figure 7). The results showed that relative mRNA expression of IL-6 and IL-1β significantly increased (*p <* 0.05), but IFN-γ, TGF-β, and IL-4 were significantly downregulated (*p <* 0.05) in the PAMK group when compared with the Control group. Besides, treatment with CTX significantly inhibited relative mRNA expression of IL-6 but significantly promoted the expression of IL-1β, IL-10, IFN-γ, TGF-β, and IL-4 (*p <* 0.05). However, the results on the combination of CTX and PAMK (CTX+PAMK group) revealed a significant difference (*p <* 0.05) from those in the CTX group and were closer to the corresponding data in the Control group.

### 2.5. Principal Component Analysis

The correlation coefficients among various indicators can be found in the Supplementary Materials. The results showed that there were 16 absolute values of the correlation coefficients greater than 0.8, accounting for 8.5% of all the results. The coefficient of correlation between RIL10 and PAL was the highest, 0.953, followed by that between RIL10 and SIL1β: 0.892. In addition, the absolute values of coefficients of correlation between SIFNγ and PN, SIL10, RIL10, and RIL6 were all greater than 0.8; the absolute values of coefficients of correlation between SIL10 and RIFNγ or STGFβ were also greater than 0.8. Therefore, SIFNγ, RIL10, and SIL10 showed the highest correlations with other indicators.

Three principal components were extracted, and eigenvalue of each principal component was greater than 1.0 (Table 1). Principal component 1 accounted for 48.59% of variance, and the three principal components had a cumulative percentage of 74.92%, which can represent variance of most of the indicators in the results.

The scores of every indicator in the three principal components (Table 2) were used to calculate the scores for different groups on each principal component (Table 3). The scores of our four groups of geese on the components suggested that CTX treatment had the greatest effect on principal components **1** and **2**, whereas PAMK had the greatest effect on component **3** (Table 3). The total score of the three components among the different groups was the highest in the CTX+PAMK group and the lowest in the CTX group, indicating that the two groups had opposite effects on the three components. In addition, scores of the CTX+PAMK group and Control group on these three components were closer, suggesting that the effects of these two treatments on the three components were similar.

**Figure 8:**
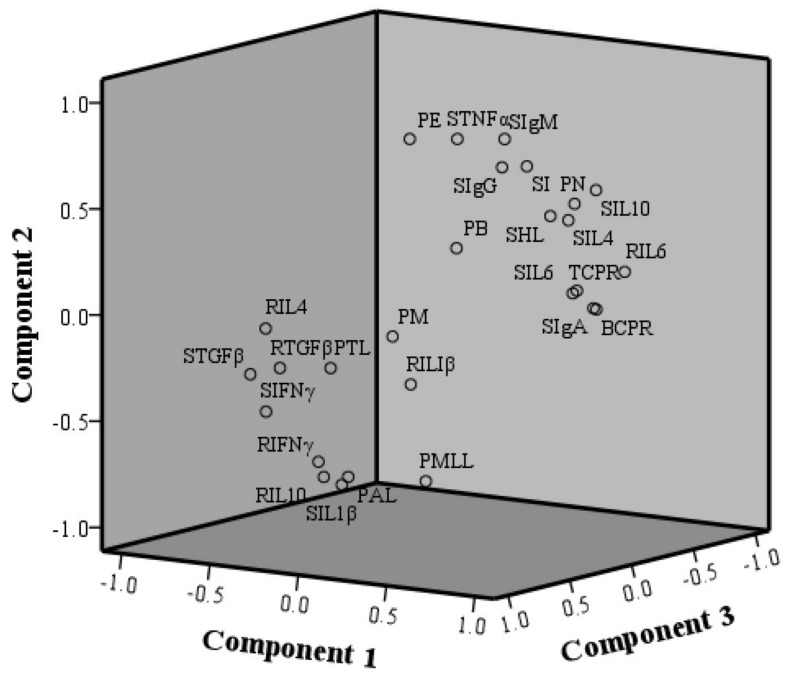
The component plot of the principal component analysis in geese.

More noticeable differences in the component matrix were detected after component rotation (Table 4). The results indicated that SIgA, TCPR, RIL6, BCPR, RTGFβ, and RIL4 play a major role in component **1**, among which SIgA, TCPR, RIL6, and BCPR were positive indicators, whereas RTGFβ and RIL4 were negative indicators. According to the scores of different indicators on the three components (Table 4), a three-dimensional component plot was constructed (Figure 8). All the indicators showed two clusters in the plot of the three principal components. Among them, humoral-immunity indicators BCPR, SIgM, SIgG, SIgA, and SHL were concentrated in one cluster, while the cellular-immunity indicators and the percentages of various leukocytes were distributed between the two clusters. The clusters of these indicators were closely related to the effects of CTX. Indicators on which CTX treatment had a promoting effect gathered into one cluster, whereas indicators on which CTX treatment had an inhibitory effect gathered into the other cluster.

## 3. Discussion

CTX is often used to set up immunosuppressive models to study immune function in mammals and poultry because of the inhibition of humoral and cellular immunity [12,13]. It has been reported that CTX can cause atrophy and weight loss of the spleen and an imbalance of various leukocytes in the peripheral blood of mice, eventually inhibiting immune function [14,15,16,17,18,19]. Therefore, a goose model of immunosuppression was prepared here via an injection of CTX, and morphology of the spleen and percentages of leukocytes were examined in this research. The results indicated that CTX decreased the spleen index and numbers of lymphocytes, increased numbers of cells with nuclear shrinkage and apoptosis, and caused aberrations in spleen morphology and an imbalance of leukocytes; these findings revealed that immunosuppression in geese was induced successfully. The reason may be that CTX inhibits the production of bone marrow hematopoietic stem cells, reduces the number of new differentiated leukocytes but also accelerates the atrophy and apoptosis of leukocytes [20]. This study found that PAMK significantly increased the CTX-inhibited spleen index, mainly by significantly increasing the spleen weight of geese.

Serum hemolysin and immunoglobulin levels and the B-cell proliferation rate mainly reflect humoral-immunity function. The level of serum hemolysin indicates the amounts of anti-sheep red blood cell antibodies produced by B cells, and directly reflects the activity of humoral immune function. Some researchers have found that traditional Chinese medicine remedies such as Yu-Ping-Feng-Powder improve the humoral immunity of mice by increasing the hemolysis of sheep red blood cells in a quantitative assay [14]. Immunoglobulins are secreted by plasma cells and have the function of neutralizing bacteria, viruses, and toxins and enhancing phagocytosis of microorganisms. It is known that CTX can reduce the number of B cells in blood and inhibit the proliferation rate of B cells cultured in vitro [21,22]; these data are similar to the results of our study. In the meantime, the decrease in B-cell number directly leads to the decrease of the number of plasma cells. This may be the reason why CTX decreases hemolysin and immunoglobulin levels. Therefore, during the cotreatment with PAMK and CTX, the decrease in the proliferation rate of B cells was significantly attenuated, and the levels of hemolysin and immunoglobulins were restored to the level of the Control group. Combined with reports that *Astragalus polysaccharides* increase the proliferation rate of B cells and serum levels of immunoglobulins (IgA, IgG, and IgM) in mice, our results confirmed that PAMK can restore humoral immune function after inhibition by CTX [15].

The T-cell proliferation rate and the levels of cytokines mainly reflect the activity of cellular immune function. Cytokines can be secreted by a variety of cells but are predominantly produced by T cells. Therefore, the reasons why the levels of cytokines in the CTX+PAMK group were restored to the levels of the Control group may be related to the proliferation rate of T cells. According to their different functions, cytokines can be subdivided into pro-inflammatory and anti-inflammatory. Pro-inflammatory cytokines are those that activate an inflammatory response, e.g., TNF-α, IFN-γ, IL-1β, and IL-6, whereas anti-inflammatory cytokines are those that inhibit the inflammatory response, for example, IL-4, IL-10, and TGF-β. In the present study, CTX mainly upregulated serum pro-inflammatory cytokines and downregulated anti-inflammatory cytokines, indicating an inflammatory process. Therefore, CTX can be used to set up a model of inflammation, such as bladder inflammation [23]. The mRNA levels of TNF-α, IFN-γ, IL-1β, IL-6, and TGF-β in the CTX group and CTX+PAMK group in the spleen were consistent with the serum levels, while concentrations of IL-4 and IL-10 were opposite to those in serum. We know that the level of mRNA expression does not exactly reflect protein expression because the translation process is regulated by microRNA or stress granules [24,25]. In the CTX+PAMK group, the levels of various cytokines in the spleen and serum were closer to those in the Control group, indicating that the effect of PAMK on cellular immunity is restoration of unbalanced cytokine levels closer to the normal levels. In the principal component analysis, we found that the relevance of humoral immune indicators is greater. In addition, scores on the three principal components showed that the results in the CTX+PAMK group and Control group are similar, and the results of the CTX group and of the CTX+PAMK group are the most distant from each other. This analysis proved that PAMK treatment can attenuate the immunosuppressive effect of CTX on the immune function in geese.

According to previous reports, the time for oral addition of PAMK to animals was 1–4 weeks [4,26,27], and no adverse effects of PAMK were reported in these studies. In addition, PAMK is one of the ingredients in traditional Chinese medicine *Atractylodes macrocephala*. Studies have shown that long-term use of *Atractylodes macrocephala* does not produce side effects, while PAMK is a safe and effective bioactive ingredient in *Atractylodes macrocephala*, so long-term use of PAMK does not produce toxic effects [28]. In addition, PAMK also has the effect of resisting immune damage and promoting immune function. This shows that adding PAMK to feeding animals has a long-term positive effect.

In conclusion, PAMK can protect the spleen from damage, stabilize the proportion of leukocytes, reverse the humoral and cellular immune dysfunction, and eventually alleviate the immunosuppression caused by CTX in geese. Besides, PAMK relieves immunosuppression in CTX-treated geese by maintaining a humoral and cellular immune balance.

## 4. Materials and Methods

### 4.1. Experimental Grouping and Treatments

All geese (*Anser cygnoides*) were treated humanely, and the experiments received prior ethical approval in accordance with Zhongkai University of Agriculture and Engineering under the approved protocol number SRM-11. Geese were purchased from Guangdong Qingyuan Jinyufeng Goose Co., Ltd., which is a professional goose-breeding company. The geese were housed in a specific pathogen-free environment and were enrolled in experiments at 1 day of age, with half of them male and half female. PAMK (purity 70%) was purchased from Tianyuan (Xi’an, China). PAMK was dissolved in 500 mL double distilled water and sprayed evenly on 5 kg of feed. After being air-dried naturally, it was thoroughly mixed with 5 kg, 10 kg, and 20 kg of feed. Finally, 40 kg of feed with a PAMK concentration of 400 mg·kg^−1^ was obtained. Two hundred geese were randomly distributed into four groups (50 per group). The four groups of geese had free access to food (including vegetables) and water. The Control and CTX groups were fed normal diets, whereas groups PAMK and CTX+PAMK were fed the normal diet supplemented with PAMK at a dose of 400 mg·kg^−1^. In addition, groups Control and PAMK were injected with 0.5 mL saline, while groups CTX and CTX+PAMK were injected with CTX (Jiangsu Hengrui Pharmaceutical, Lianyungang, China) at 40 mg·(kg body weight)^−1^ per day starting at 12–14 days of age, once daily at the same time. Spleens and blood were collected at 28 days of age. All the organs were placed in liquid nitrogen immediately and stored at −80 °C until analysis.

### 4.2. The Spleen Index Assay

The weights of spleens were measured immediately after the geese were euthanized. The spleen index is the ratio of spleen weight (g) to body weight (kg).

### 4.3. Spleen Histology and Ultramicroscopic Morphology Observation

HE staining: The paraffin-fixed blocks were serially sectioned into 5- to 6-μm-thick coronal slices. For routine histological examination, the paraffin sections were stained with HE. HE-stained slices were analyzed under a Nikon fluorescence microscope (Nikon, Tokyo, Japan).

TEM: Each spleen was divided into small blocks of 1 mm^3^ and then fixed with 2.5% glutaraldehyde at 4 °C. Ultrathin slices with 50–70 nm thickness were prepared and stained with uranyl acetate and lead citrate following the conventional protocol. The samples were examined under a transmission electron microscope (JEM-1400, Tokyo, Japan).

SEM: The spleens were fixed with 2.5% glutaraldehyde for >2 h and observed under a scanning electron microscope (Hitachi S3000N, Tokyo, Japan).

### 4.4. Leukocyte Counting

EDTA was applied to prevent coagulation of blood. The collected blood was gently mixed and placed dropwise onto a slide to prepare a blood smear. After the blood smears were air dried, they were stained according to the instruction manual for Wright’s stain. The blood smears were examined under a light microscope, and 200 leukocytes were selected from 5 fields of vision. The percentages of neutrophils, eosinophils, basophils, monocytes, lymphocytes (medium and large size), atypical lymphocytes, and total lymphocytes among the 200 leukocytes were calculated separately.

### 4.5. T-Cell and B-Cell Proliferation Assays

Spleens were collected in the four groups and the procedure of isolation of lymphocytes was similar to the one published by Cao [29]. PHA (15 μg·mL^−1^; Sigma, St. Louis, MO, USA) and LPS (25 μg·mL^−1^; Sigma, St. Louis, MO, USA) were added into the culture medium to activate T-cell and B-cell proliferation, respectively. After cultivation for 44 h, an MTS Kit (Promega, Madison, WI, USA) was used to measure the proliferation rate. The ratio of the absorbance of cells treated with PHA to the absorbance without PHA served as an indicator of the T-cell proliferation rate. The ratio of the absorbance of cells treated with LPS to the absorbance without LPS was an indicator of the B-cell proliferation rate.

### 4.6. Assays of Serum Cytokines and Immunoglobulins

Venous blood was collected to separate serum. Concentrations of IgA, IgG, IgM, IL-1β, TNF-α, IFN-γ, TGF-β, IL-4, IL-6, and IL-10 in serum were measured by means of enzyme-linked immunosorbent assay (ELISA) kits (Nanjing Jiancheng Bioengineering Institute, Nanjing, China).

### 4.7. The Serum Hemolysin Assay

Eight geese with similar body weight were selected in 4 groups. At 21 days of age, a 5% chicken red blood cell suspension (Hongquan, Guangzhou, China) was employed as an immunogen and injected intraperitoneally. Seven days later, serum of geese was separated after fasting for 6 h and incubated in a 56 °C water bath for 30 min to inactivate complement. Serum was diluted 50-fold with physiological saline. After that, 1 mL of diluted serum, 0.5 mL of fresh guinea pig serum (Hongquan, Guangzhou, China) as a complement, and 0.5 mL of chicken red blood cells were mixed and incubated at 37 °C for 30 min. The optical density at 540 nm of the supernatant was measured on a spectrophotometer as a metric of hemolysin concentration.

### 4.8. Quantitative Reverse-Transcription PCR Analysis

Specific primers matching reverse-transcribed mRNA were designed (Table 5). The procedure for quantitative PCR was similar to the one published by Yao [30].

### 4.9. Principal Component Analysis

From the data, we subtracted the mean value and divided the result by the standard deviation to obtain the standardized data and make the standardized data comparable and compliant with the normal distribution. In the SPSS statistical software, the dimensionality of data was reduced to obtain the correlation matrix of all indicators. The first three principal components with the largest extraction rate were obtained. According to the scores in Table 2, a linear combination of the first three principal components and 27 indicators was obtained. The scores of each group on every principal component were calculated. The factor variance is greatly affected by orthogonal rotation, so that the original variables in the public factors undergo polarization. The main component of the rotation matrix is closer to 1 or closer to 0; therefore, a principal component can be better explained.

### 4.10. Statistics

The results were expressed as means ± SD of the indicated number of experiments. All the quantitative PCRs were run in triplicate, and the relative expression levels were measured in terms of threshold cycle (Ct) values and were normalized via the formula 2^−∆∆Ct^. Statistical analysis of all data was performed in SPSS for Windows (version 18, SPSS Inc, Chicago, IL, USA). When the main effect (*p <* 0.05) was identified by one-way analysis of variance, comparisons of means were made next. Differences between means were assessed by Tukey’s honestly significant difference test.

## Figures and Tables

**Table 1:** Principal components and extracted sums of variance measures in geese.

Component	Eigenvalue	% of Variance	Cumulative %
1	13.119	48.589	48.589
2	3.888	14.398	62.988
3	3.222	11.934	74.922

**Table 2:** The matrix of component scores.

Indicators	Abbreviation	Component 1	Component 2	Component 3
Spleen index	SI	0.002	0.071	0.034
Percentage of neutrophil	PN	0.072	0.020	−0.020
Percentage of eosinophils	PE	−0.009	0.222	0.205
Percentage of basophils	PB	0.159	−0.079	−0.037
Percentage of monocytes	PM	−0.024	0.081	−0.044
Percentage of medium and large lymphocytes	PMLL	0.168	0.008	0.149
Percentage of total lymphocytes	PTL	0.149	−0.081	0.024
Percentage of atypical lymphocytes	PAL	−0.032	0.236	0.251
Serum hemolysin level	SHL	−0.103	0.032	0.066
Serum IgM level	SIgM	0.089	−0.067	−0.021
Serum IgA level	SIgA	−0.008	0.019	−0.160
Serum IgG level	SIgG	0.036	0.004	−0.142
B-cell proliferation rate	BCPR	−0.039	0.035	0.161
T-cell proliferation rate	TCPR	0.028	−0.107	0.046
Serum TNF-α level	STNFα	−0.031	−0.035	−0.250
Serum TGF-β level	STGFβ	−0.109	0.116	−0.057
Serum IL-6 level	SIL6	0.005	−0.058	0.041
Serum IL-4 level	SIL4	0.021	−0.086	−0.093
Serum IL-10 level	SIL10	0.105	−0.176	−0.037
Serum IFN-γ level	SIFNγ	0.058	0.134	0.391
Serum IL-1β level	SIL1β	0.010	−0.158	−0.050
Relative mRNA expression of TGF-β	RTGFβ	−0.110	−0.051	−0.071
Relative mRNA expression of IL-6	RIL6	0.140	−0.088	−0.038
Relative mRNA expression of IL-4	RIL4	−0.158	0.068	−0.015
Relative mRNA expression of IL-10	RIL10	0.003	−0.134	−0.007
Relative mRNA expression of IFN-γ	RIFNγ	0.023	−0.053	0.116
Relative mRNA expression of IL-1β	RILIβ	−0.060	−0.042	−0.100

**Table 3:** Scores of the four groups of geese on three principal components.

Groups	Component 1	Component 2	Component 3	Total	Ranking
Control	−0.177	0.942	0.461	1.226	2
PAMK	0.513	0.374	−1.348	−0.462	3
CTX	−1.385	−1.249	0.117	−2.516	4
CTX+PAMK	0.965	−0.116	0.746	1.594	1

**Table 4:** The rotated component matrix.

Indicators	Component 1 (48.59%)	Component 2 (14.40%)	Component 3 (11.93%)
SI	0.319	0.644	−0.284
SHL	0.563	0.457	−0.125
SIgM	0.37	0.814	−0.03
SIgA	0.856	0.062	−0.055
SIgG	0.184	0.627	−0.275
TCPR	0.916	0.175	0.198
BCPR	0.82	0.042	−0.131
STNFα	0.191	0.815	0.097
STGFβ	−0.781	−0.351	0.383
SIL6	0.581	0.08	−0.314
SIL4	0.323	0.342	−0.614
SIL10	0.538	0.518	−0.531
SIFNγ	−0.529	−0.468	0.616
SIL1β	−0.265	−0.819	0.379
PN	0.214	0.379	−0.819
PE	−0.291	0.722	−0.207
PB	0.022	0.249	−0.138
PM	−0.109	−0.133	0.192
PMLL	0.138	−0.777	0.275
PTL	0.057	−0.158	0.929
PAL	−0.36	−0.819	0.192
RTGFβ	−0.809	−0.365	0.103
RIL6	0.857	0.198	−0.306
RIL4	−0.893	−0.188	0.099
RIL10	−0.418	−0.809	0.307
RIFNγ	−0.285	−0.69	0.54
RILIβ	−0.325	−0.447	−0.265

**Table 5:** Primer sequences for quantitative PCR.

cDNA	Primer Sequence (5′→3′)
IFNG-F	CCAGATTGTTTCCCTGTACTTG
IFNG-R	CATCAGAAAGGGTGTCTCTCA
IL1β-F	ACGGTGTGGGGACATTCATC
IL1β-R	AGGCGAAGCTTCTTCTGTGG
IL4-F	GGCATCTACCTCAACTTGCT
IL4-R	CTCTTTCGCTACTCGTTGGA
IL6-F	ACGATAAGGCAGATGGTGAT
IL6-R	TCCAGGTCTTATCCGACTTC
IL10-F	ATCATGACATGGACCCGGTA
IL10-R	ATTGCTCCATGACAGTTGCT
TGF-β-F	CATCACAGAGACAGGAACCTT
TGF-β-R	CTTTCACATCACCACTGGAA
β-actin-F	GCACCCAGCACGATGAAAAT
β-actin-R	GACAATGGAGGGTCCGGATT

## Supplementary Materials

The following are available online. Figure S1: The correlation coefficients among various indicators.

## References

[1] Sun W., Meng K., Qi C., Yang X., Wang Y., Fan W., Yan Z., Zhao X., Liu J. (2015). Immune-enhancing activity of polysaccharides isolated from *Atractylodis macrocephalae* Koidz. Carbohydr. Polym..

[2] Xu D., Li B., Cao N., Li W., Tian Y., Huang Y. (2017). The protective effects of polysaccharide of *Atractylodes macrocephala* Koidz (PAMK) on the chicken spleen under heat stress via antagonizing apoptosis and restoring the immune function. Oncotarget.

[3] Son Y.O., Kook S.H., Lee J.C. (2017). Glycoproteins and Polysaccharides are the Main Class of Active Constituents Required for Lymphocyte Stimulation and Antigen-Specific Immune Response Induction by Traditional Medicinal Herbal Plants. J. Med. Food.

[4] Liu J., Chen X., Yue C., Hou R., Chen J., Lu Y., Li X., Li R., Liu C., Gao Z. (2015). Effect of selenylation modification on immune-enhancing activity of *Atractylodes macrocephala* polysaccharide. Int. J. Biol. Macromol..

[5] Xu D., Tian Y. (2015). Selenium and Polysaccharides of *Atractylodes macrocephala* Koidz Play Different Roles in Improving the Immune Response Induced by Heat Stress in Chickens. Biol. Trace Elem. Res..

[6] Zhou L., Liu Z., Wang Z., Yu S., Long T., Zhou X., Bao Y. (2017). Astragalus polysaccharides exerts immunomodulatory effects via TLR4-mediated MyD88-dependent signaling pathway in vitro and in vivo. Sci. Rep..

[7] Zhang J.L., Huang W.M., Zeng Q.Y. (2015). Atractylenolide I protects mice from lipopolysaccharide-induced acute lung injury. Eur. J. Pharmacol..

[8] Lee S.J., Kim J.J., Kang K.Y., Hwang Y.H., Jeong G.Y., Jo S.K., Jung U., Park H.R., Yee S.T. (2016). Herbal preparation (HemoHIM) enhanced functional maturation of bone marrow-derived dendritic cells mediated toll-like receptor 4. BMC Complement. Altern. Med..

[9] Zhao T., Feng Y., Li J., Mao R., Zou Y., Feng W., Zheng D., Wang W., Chen Y., Yang L. (2014). Schisandra polysaccharide evokes immunomodulatory activity through TLR 4-mediated activation of macrophages. Int. J. Biol. Macromol..

[10] Angel-Morales G., Noratto G., Mertens-Talcott S.U. (2012). Standardized curcuminoid extract (*Curcuma longa* L.) decreases gene expression related to inflammation and interacts with associated microRNAs in human umbilical vein endothelial cells (HUVEC). Food Funct..

[11] Ji G.-Q., Chen R.-Q., Zheng J.-X. (2014). Macrophage activation by polysaccharides from *Atractylodes macrocephala* Koidz through the nuclear factor-κB pathway. Pharm. Biol..

[12] Guo L., Liu J., Hu Y., Wang D., Li Z., Zhang J., Qin T., Liu X., Liu C., Zhao X. (2012). Astragalus polysaccharide and sulfated epimedium polysaccharide synergistically resist the immunosuppression. Carbohydr. Polym..

[13] Wang M., Meng X.Y., Yang R.L., Qin T., Wang X.Y., Zhang K.Y., Fei C.Z., Li Y., Hu Y., Xue F.Q. (2012). Cordyceps militaris polysaccharides can enhance the immunity and antioxidation activity in immunosuppressed mice. Carbohydr. Polym..

[14] Chen X.T., Li J., Wang H.L., Cheng W.M., Zhang L., Ge J.F. (2006). Immunomodulating effects of fractioned polysaccharides isolated from Yu-Ping-Feng-Powder in cyclophosphamide-treated mice. Am. J. Chin. Med..

[15] Meng F., Xu P., Wang X., Huang Y., Wu L., Chen Y., Teng L., Wang D. (2017). Investigation on the immunomodulatory activities of *Sarcodon imbricatus* extracts in a cyclophosphamide (CTX)-induced immunosuppressanted mouse model. Saudi Pharm. J..

[16] Ikezawa Y., Nakazawa M., Tamura C., Takahashi K., Minami M., Ikezawa Z. (2005). Cyclophosphamide decreases the number, percentage and the function of CD25^+^ CD4^+^ regulatory T cells, which suppress induction of contact hypersensitivity. J. Dermatol. Sci..

[17] Siracusa F., Alp Ö.S., Maschmeyer P., McGrath M., Mashreghi M.-F., Hojyo S., Chang H.-D., Tokoyoda K., Radbruch A. (2017). Maintenance of CD8^+^ memory T lymphocytes in the spleen but not in the bone marrow is dependent on proliferation. Euro. J. Immunol..

[18] Tanahashi T., Sekiguchi N., Matsuda K., Matsumoto A., Ito T., Nakazawa H., Ishida F. (2016). A screening method with lymphocyte percentage and proportion of granular lymphocytes in the peripheral blood for large granular lymphocyte (LGL) leukemia. Int. J. Hematol..

[19] Wang J., Wang J., Gao K., Ye X., Lei W., Jin T., Dong L., Xuan Q., Zhang Z., Feng M. (2017). Lymphocyte-to-monocyte ratio is associated with prognosis of diffuse large B-cell lymphoma_correlation with CD163 positive M2 type tumor-associated macrophages, not PD-1 positive tumor-infiltrating lymphocytes. Oncotarget.

[20] Sun C., Yang J., Pan L., Guo N., Li B., Yao J., Wang M., Qi C., Zhang G., Liu Z. (2017). Improvement of icaritin on hematopoietic function in cyclophosphamide-induced myelosuppression mice. Immunopharmacol. Immunotoxicol..

[21] Huang R., Zhang J., Liu Y., Hao Y., Yang C., Wu K., Cao S., Wu C. (2013). Immunomodulatory effects of polysaccharopeptide in immunosuppressed mice induced by cyclophosphamide. Mol. Med. Rep..

[22] Wan C.P., Gao L.X., Hou L.F., Yang X.Q., He P.L., Yang Y.F., Tang W., Yue J.M., Li J., Zuo J.P. (2013). Astragaloside II triggers T cell activation through regulation of CD45 protein tyrosine phosphatase activity. Acta Pharmacol. Sin..

[23] Hughes F.M., Vivar N.P., Kennis J.G., Pratt-Thomas J.D., Lowe D.W., Shaner B.E., Nietert P.J., Spruill L.S., Purves J.T. (2014). Inflammasomes are important mediators of cyclophosphamide-induced bladder inflammation. Am. J. Physiol. Renal Physiol..

[24] Wang F., Lu J., Peng X., Wang J., Liu X., Chen X., Jiang Y., Li X., Zhang B. (2016). Integrated analysis of microRNA regulatory network in nasopharyngeal carcinoma with deep sequencing. J. Exp. Clin. Cancer Res..

[25] Khong A., Matheny T., Jain S., Mitchell S.F., Wheeler J.R., Parker R. (2017). The Stress Granule Transcriptome Reveals Principles of mRNA Accumulation in Stress Granules. Mol. Cell.

[26] Xu D., Li W., Huang Y., He J., Tian Y. (2014). The effect of selenium and polysaccharide of *Atractylodes macrocephala* Koidz. (PAMK) on immune response in chicken spleen under heat stress. Biol. Trace Elem. Res..

[27] Jin C., Zhang P.J., Bao C.Q., Gu Y.L., Xu B.H., Li C.W., Li J.P., Bo P., Liu X.N. (2011). Protective effects of *Atractylodes macrocephala* polysaccharide on liver ischemia-reperfusion injury and its possible mechanism in rats. Am. J. Chin. Med..

[28] Wang X., Li L., Ran X., Dou D., Li B., Yang B., Li W., Koike K., Kuang H. (2016). What caused the changes in the usage of Atractylodis Macrocephalae Rhizoma from ancient to current times?. J. Nat. Med..

[29] Cao N., Li W., Li B., Tian Y., Xu D. (2017). Transcriptome profiling reveals the immune response of goose T cells under selenium stimuli. Anim. Sci. J..

[30] Yao H.D., Wu Q., Zhang Z.W., Li S., Wang X.L., Lei X.G., Xu S.W. (2013). Selenoprotein W serves as an antioxidant in chicken myoblasts. Biochim. Biophys. Acta.

