# Supplementary material for: Polysaccharide of Atractylodes macrocephala Koidz (PAMK) Relieves Immunosuppression in Cyclophosphamide-Treated Geese by Maintaining a Humoral and Cellular Immune Balance
Source: Molecules. 2018 Apr 17;23(4):932. doi: 10.3390/molecules23040932 (PMC6017956; doi:10.3390/molecules23040932)
Supplement: Supplementary file 1 [file molecules-23-00932-s001.zip › molecules-282775-SI.pdf]

Appendix

	SI	PN	PE	PB	PM	PMLL	PTL	PAL	SHL	SIgM	SIgA	SIgG	BCPR	TCPR	STNFα	STGFβ	SIL6	SIL4	SIL10	SIFNγ	SIL1β	RTGFβ	RIL6	RIL4	RIL10	RIFNγ	RILIβ
SI	1.000																										
PN	0.564	1.000																									
PE	0.366	0.228	1.000																								
PB	0.493	0.304	0.350	1.000																							
PM	-0.307	-0.503	0.319	-0.170	1.000																						
PMLL	-0.581	-0.501	-0.675	-0.380	0.240	1.000																					
PTL	-0.405	-0.869	-0.289	-0.299	0.212	0.425	1.000																				
PAL	-0.569	-0.539	-0.609	-0.238	0.019	0.556	0.257	1.000																			
SHL	0.546	0.239	0.343	0.075	0.078	-0.118	-0.045	-0.615	1.000																		
SIgM	0.702	0.519	0.336	0.280	-0.395	-0.525	-0.158	-0.794	0.531	1.000																	
SIgA	0.260	0.181	-0.106	-0.162	0.067	0.169	0.025	-0.388	0.588	0.338	1.000																
SIgG	0.615	0.413	0.553	0.177	-0.101	-0.490	-0.312	-0.534	0.609	0.468	0.319	1.000															
BCPR	0.368	0.394	-0.221	0.252	-0.310	-0.075	-0.116	-0.343	0.332	0.380	0.599	0.148	1.000														
TCPR	0.274	0.069	-0.122	-0.015	-0.022	-0.045	0.224	-0.447	0.506	0.407	0.778	0.214	0.744	1.000													
STNFα	0.611	0.388	0.387	0.327	-0.202	-0.577	-0.184	-0.662	0.295	0.799	0.156	0.457	0.209	0.290	1.000												
STGFβ	-0.595	-0.656	-0.055	-0.199	0.347	0.291	0.391	0.628	-0.652	-0.607	-0.688	-0.451	-0.732	-0.687	-0.416	1.000											
SIL6	0.442	0.559	-0.236	0.289	-0.505	-0.076	-0.343	-0.260	0.363	0.483	0.348	0.265	0.521	0.376	0.298	-0.635	1.000										
SIL4	0.522	0.744	0.086	0.039	-0.410	-0.438	-0.562	-0.447	0.360	0.531	0.172	0.339	0.409	0.265	0.278	-0.559	0.563	1.000									
SIL10	0.575	0.737	0.359	0.204	-0.243	-0.510	-0.522	-0.737	0.554	0.629	0.545	0.482	0.601	0.506	0.430	-0.819	0.387	0.620	1.000								
SIFNγ	-0.716	-0.836	-0.270	-0.363	0.285	0.438	0.635	0.717	-0.558	-0.663	-0.465	-0.487	-0.510	-0.391	-0.465	0.786	-0.655	-0.718	-0.813	1.000							
SIL1β	-0.707	-0.646	-0.716	-0.334	0.063	0.648	0.473	0.875	-0.646	-0.775	-0.349	-0.670	-0.298	-0.286	-0.665	0.598	-0.315	-0.496	-0.791	0.761	1.000						
RTGFβ	-0.430	-0.436	0.046	0.012	0.217	0.301	0.122	0.622	-0.454	-0.549	-0.624	-0.312	-0.720	-0.844	-0.478	0.797	-0.510	-0.537	-0.674	0.668	0.462	1.000					
RIL6	0.551	0.587	-0.079	0.336	-0.271	-0.145	-0.335	-0.526	0.536	0.557	0.691	0.293	0.776	0.733	0.413	-0.859	0.749	0.553	0.664	-0.802	-0.510	-0.787	1.000				
RIL4	-0.500	-0.295	0.032	-0.164	0.061	0.171	0.099	0.434	-0.543	-0.405	-0.736	-0.339	-0.725	-0.886	-0.330	0.751	-0.516	-0.407	-0.649	0.621	0.433	0.841	-0.834	1.000			
RIL10	-0.713	-0.640	-0.540	-0.236	0.137	0.571	0.362	0.953	-0.707	-0.809	-0.426	-0.648	-0.405	-0.439	-0.672	0.739	-0.361	-0.555	-0.795	0.809	0.892	0.671	-0.599	0.499	1.000		
RIFNγ	-0.746	-0.750	-0.561	-0.363	0.249	0.769	0.615	0.712	-0.480	-0.613	-0.334	-0.642	-0.347	-0.316	-0.588	0.689	-0.293	-0.599	-0.824	0.789	0.812	0.546	-0.570	0.509	0.799	1.000	
RILIβ	-0.315	0.204	-0.551	-0.156	-0.586	0.200	-0.202	0.484	-0.629	-0.273	-0.455	-0.429	-0.103	-0.437	-0.348	0.237	0.090	0.200	-0.274	0.178	0.519	0.241	-0.219	0.432	0.432	0.300	1.000
